# Providing peer navigation services to women with a history of opioid misuse pre- and post-release from jail: A program description

**DOI:** 10.1017/cts.2022.441

**Published:** 2022-08-10

**Authors:** Martha Tillson, Amanda Fallin-Bennett, Michele Staton

**Affiliations:** 1 University of Kentucky Center on Drug and Alcohol Research, Lexington, KY, USA; 2 University of Kentucky College of Arts and Sciences, Department of Sociology, Lexington, KY, USA; 3 University of Kentucky College of Nursing, Lexington, KY, USA; 4 Voices of Hope, Lexington, KY, USA; 5 University of Kentucky College of Medicine, Department of Behavioral Science, Lexington, KY, USA

**Keywords:** Criminal justice system, peer navigation, women, opioid use disorder, treatment access, program description, telehealth

## Abstract

**Background:**

Justice system-involved women with opioid use disorder (OUD) experience layered health risks and stigma, yet peer navigation services during reentry may support positive outcomes. This manuscript offers a program description of a women’s peer navigation intervention delivered pre- and post-release from jail to remove barriers to women’s access to OUD treatment, including medications for opioid use disorder (MOUD).

**Methods:**

All data were collected as part of a NIH/NIDA-funded national cooperative, the Justice Community Opioid Innovation Network (JCOIN) project. Through the larger study’s intervention, women in jail with OUD are connected via videoconference to a peer navigator, who provides an initial reentry recovery assessment and 12+ weeks of recovery support sessions post-release. Qualitative analyses examined peers’ notes from initial sessions with women (N = 50) and in-depth interviews with peers (N = 3).

**Results:**

Peers’ notes from initial sessions suggest that women anticipate challenges to successful recovery and community reentry. More than half of women (51.9%) chose OUD treatment as their primary goal, while others selected more basic needs (e.g. housing, transportation). In qualitative interviews, peers described women’s transitions to the community as unpredictable, creating difficulties for reentry planning, particularly for rural women. Peers also described challenges with stigma against MOUD and establishing relationships via telehealth, but ultimately believed their role was valuable in providing resource referrals, support, and hope for recovery.

**Conclusions:**

For women with OUD, peer navigation can offer critical linkages to services at release from jail, in addition to hope, encouragement, and solidarity. Findings provide important insights for future peer-based interventions.

Women face numerous barriers to successful community reentry following incarceration, including challenges obtaining housing, employment, prosocial support networks, and – critically – access to preventive services and treatment for physical or mental health problems [1,2]. These challenges are exacerbated for women with a history of substance misuse, particularly those with opioid use disorder (OUD), who may experience further co-occurring health issues [3] as well as a heightened risk of overdose during the immediate post-release period [4]. After community reentry, women must navigate intersecting stigmas of past incarceration and OUD and may also lack access to/knowledge of resources, support, and encouragement to achieve their goals [5]. This may limit women’s capacity to connect to evidence-based treatment, including medications for opioid use disorder (MOUD).

Peer-based services have demonstrated promise in supporting positive health outcomes for individuals seeking recovery from substance misuse [6–8]. Broadly, “peers” are individuals with lived experience (e.g. incarceration, mental health issues, and/or substance misuse) working in trained/certified staff positions to address a variety of needs, including advocacy, community-building, role-modeling, resource-gathering, or motivational support [9]. Through a combination of professional training and lived experience, peers can bridge communication gaps between clinicians and clients and speak with legitimacy grounded in either/both the service user or trained specialist role [10,11]. For individuals with overlapping histories of substance misuse and justice system involvement, peers who share such intersectional lived experiences can more easily establish trust and rapport [12–14], particularly among women [15,16], and support individuals to remain unincarcerated [17,18].

“Peer navigators” serve these support and advocacy functions, but additionally act as linkage facilitators to connect individuals to services and support treatment engagement and retention. Peer navigators can be seen as an extension of a clinical care team, much like community health workers, and can play key roles in engaging individuals in research and linkage to care [19–21]. Thus, peer navigation has the potential to improve the health and well-being of women with OUD post-incarceration through sharing of professional and experiential knowledge related to systems of care for OUD and related health issues.

Peer navigation interventions additionally have the benefit of expanding community outreach and engagement opportunities, aligning with national goals and objectives for translational science [22] and establishing/expanding relationships between researchers, recovery communities, and substance use treatment providers. At the point of reentry from incarceration, peer navigation may also build or strengthen relationships between criminal justice and healthcare systems, creating more sustainable pathways for access to treatment and care. These pathways may be particularly beneficial for women in jails, who are typically incarcerated for shorter periods, with less access to services, compared to men [23]. Additionally, for women returning to rural communities where services may be geographically dispersed with fewer transportation resources available [24], linkage facilitation interventions may be especially valuable, addressing barriers to treatment in a targeted manner.

## Current Study

Although little research has been devoted to the integration of peer support specialists for substance use recovery in criminal justice settings, studies have demonstrated that the inclusion of individuals with lived experiences of *both* incarceration and addiction is valuable when serving similar populations [12–14] and can support positive outcomes [17,18]. Specific to OUD, peers can also help justice system-involved individuals to manage and overcome MOUD stigma [25]. However, the research base examining *peer navigation* as a specific subcategory of peer recovery support services for justice-involved individuals is extremely limited, and little is known about how such services can best support justice-involved women with OUD as a unique demographic. Descriptive, process-level data from current interventions and programs are an important step towards expanding this emerging area of research and documenting potential challenges and benefits of implementing such programs to support women’s recovery and reentry in a real-world setting. The present study has incorporated a peer navigation intervention for women with OUD both pre- and post-release from jail and has just completed its first year of in-the-field implementation. Thus, the present paper aims to (1) outline goals, supports, and barriers to treatment access identified by incarcerated women with OUD before release from jail; (2) describe women’s transition to the community from the perspectives of peer navigators; and (3) discuss intervention challenges, successes, and lessons learned identified by peer navigators during the first year of study implementation.

## Materials and Methods

### Participants

As part of a NIH/NIDA-funded research cooperative, the Justice Community Opioid Innovation Network (JCOIN) project (UG1DA050069), the present study has implemented a women’s peer navigation intervention delivered pre- and post-release from jail, with the goal of increasing women’s access, initiation, and engagement with MOUD [26]. Women are screened by research staff via videoconference or in-person in a private room at the jail and are eligible to be included in the study if they: 1) screen positive for OUD on the Diagnostic Statistical Manual (DSM-5) [27] checklist (2+ criteria) or the NIDA-modified Alcohol, Smoking, and Substance Involvement Screening Test (NM-ASSIST; [28] score of 4+ for street or prescription opioids), 2) are willing to participate in MOUD pretreatment, 3) have no evidence of cognitive impairment or active psychosis, and 4) have an anticipated release date between 7 and 60 days from screening.

### Procedures

Women are randomly selected from rosters at six jail sites (as of February 14, 2022) for the larger clinical trial, including two comparison and four experimental sites. Three of the four counties where experimental jail sites were located are classified as “nonmetro” using the U.S. Department of Agriculture’s Rural-Urban Continuum Codes (i.e.,“Beale codes”) [29]. Eligible, consenting women at experimental sites complete a baseline interview with research staff and are randomized to receive either 1) a MOUD pretreatment telehealth session with a community provider, or 2) MOUD pretreatment telehealth plus peer navigation (see Staton et al. [26] for a full description of study design and intervention conditions). Pretreatment telehealth intake sessions include a psychosocial assessment and MOUD education. Women randomized to receive peer navigation services are also connected via videoconference to a peer from a partnering recovery community organization for an initial assessment and reentry recovery planning. Consistent with the study aims and participant population, all peers identify as women in recovery from OUD, have a history of justice system involvement (e.g. arrests and/or incarceration), and/or have previously been prescribed MOUD.

Upon women’s release from jail, peers attempt to reconnect with women using locator information provided in the initial session and continue to offer at least 12 weeks of weekly recovery support sessions via phone. All tracking and locating efforts are logged in spreadsheets and notes from each baseline and follow-up session are recorded in REDCap using a series of standardized forms (forms available upon request).

To describe the peer navigation program and activities during the first year of implementation (February 2021–February 2022), a review was conducted of information collected by peers during initial reentry recovery assessments with women (N = 52), either via videoconference while incarcerated (n = 48) or by phone shortly following release from jail (n = 4), for women who were released prior to their initial assessment. Reentry recovery assessment aims include introduction/rapport building, orientation to peer navigation services, discussion of the participants’ barriers and facilitating factors for recovery, post-release goal setting, and assessment of recovery capital. Content analysis of REDCap session data was used to outline goals, supports/resources, and barriers identified by intervention participants during their initial peer navigation sessions.

In addition to session-by-session records, in-depth interviews were performed with all peers responsible for intervention delivery during the first year of implementation (N = 3) to provide additional process-level context for session-by-session records, to document peers’ perspectives on participants’ transition to the community, and to discuss intervention challenges, successes, and lessons learned identified during the first year. Peers completed 60-minute videoconference interviews and were compensated with a small University promotional item. All interviews were recorded, transcribed verbatim, and de-identified. All data collection procedures were protected under a federal Certificate of Confidentiality and approved by the University Institutional Review Board.

### Measures

#### Reentry recovery assessments with incarcerated women with OUD

Initial assessments conducted by peers included the following elements.

##### Reentry recovery domains

During the initial reentry recovery assessment, peers used a standardized form to evaluate the participant’s goals, supports/resources, and barriers in the following areas: recovery (support, meetings, treatment, and MOUD), health (mental and physical), relationships (family, children, and friends), spirituality, transportation, career, and education. Information was entered in open-response form, with fields skipped if the domain was not discussed.

##### Primary post-release goal

Peers also documented the participant’s primary goal post-release, including steps and resources needed, how the participant will know when they have reached their goal, when they want to reach the goal by, and why the goal is important to them (all using open-response fields).

##### Recovery capital

Recovery capital was measured using the Brief Assessment of Recovery Capital (BARC-10), a 10-item validated measure of the participant’s psychological, physical, social, and environmental resources to mitigate the stress burden of achieving recovery, measured as a unified construct [30]. Example items include “There are more important things to me in life than using substances” and “In general I am happy with my life.” Responses are recorded on a 5-point Likert scale from “strongly disagree” (=1) to “strongly agree” (=5).

#### Qualitative interviews with peer navigators

In the qualitative interviews, peers were asked to reflect on participants’ goals, barriers, and needs identified pre-release; participants’ interest or beliefs about MOUD; and challenges and successes experienced by women post-release.

### Analytic Plan

To describe reentry recovery domains, a conventional qualitative content analysis [31] was used to categorize participant responses into subcategories beneath each domain heading (e.g. spirituality, transportation, or career). Specifically, for each domain, responses were reviewed and a basic schema of two to five codes was developed. Schemas were developed and codes applied by the first author (MT) and reviewed by one of the paper’s coauthors (AFB) to resolve any discrepancies in interpretation or coding. An identical process was used to classify participants’ primary goals, as recorded on the goal setting form. Frequencies in each coded category were calculated and presented as proportions. For the BARC-10, item responses were summed to create an overall scale score of 10–50, with higher scores indicating a greater degree of recovery capital, and descriptive statistics were calculated.

Directed content analysis was performed to analyze qualitative interview data [31], with coding guided by the aims of the paper. Sections of transcripts were identified in which the interviewer asked peers to discuss 1) the initial reentry recovery assessment completed with study participants, and what they perceived as women’s most critical needs when preparing for reentry; 2) challenges they had experienced in delivering the peer navigation intervention to study participants; and 3) successes and lessons learned during the first year of study implementation. These sections were reviewed by the first author (MT) to identify primary themes, which were reviewed and discussed with the paper’s coauthors (MS, AFB) for clarity and accuracy. Peers who were interviewed were also offered the opportunity to review the written results section to provide corrections or additional insight. Selected representative quotes are presented for each section but are not attributed to individual peers to protect anonymity given the small number of peers working with the study.

## Results

### Reentry Recovery Assessments with Incarcerated Women with OUD

Initial reentry recovery assessments completed with study participants (N = 52) ranged from 15 to 45 min, with an average of 26.4 (*SD* = 6.5). According to baseline interviews conducted by research study staff, participants who completed an initial reentry recovery assessment with a peer were on average 36.5 years old (range 21–57), 88.5% non-Hispanic white, 71.2% heterosexual, and had been incarcerated for a median of 75.5 days (range 7–690). As shown in Table 1, peers asked women to discuss needs/barriers, resources/supports, and additional goals, across a variety of life domains relating to recovery capital.

**Table 1.** Reentry recovery assessment needs/barriers and resources/supports (N = 52)

	%	***n***
**Recovery**		
*Recovery supports*		
Family	53.8%	28
“Good support”	15.4%	8
No support	13.5%	7
*Mutual support meetings*		
12-step fellowships	73.1%	38
Religious/faith-based meetings	7.7%	4
Unsure about meetings/did not want to go	5.8%	3
*OUD treatment*		
Residential treatment	50.0%	26
Medications for opioid use disorder (MOUD)	17.3%	9
Outpatient/intensive outpatient	13.5%	7
*Medications for opioid use disorder (MOUD; n = 29)* *		
Extended-release naltrexone (Vivitrol®)	44.8%	13
Buprenorphine/naloxone (Suboxone®)	31.0%	9
Methadone	13.8%	4
No MOUD	10.3%	3
**Health**		
*Mental health*		
Wants to start/continue medications	53.8%	28
Mental health good/stable	23.1%	12
Mental health not good	7.7%	4
*Physical health*		
Good health	46.2%	24
Overall good health, but areas to work on	13.5%	7
Have medical issues	30.8%	16
**Relationships**		
*Family*		
Good and/or currently improving	57.7%	30
Mixed (both good and bad)	30.8%	16
Poor/no family support	5.8%	3
Children? *(n = 29*)*		
No children	20.7%	6
Mentioned children	48.3%	14
Not living with children/no custody	27.6%	8
*Friends*		
At least one sober friend	44.2%	23
No sober friends	46.2%	24
**Spirituality**		
Is religious/spiritual	67.3%	35
“Working on” spirituality	15.4%	8
“Unsure” about spirituality	7.7%	4
Not spiritual	5.8%	3
**Transportation**		
Has own car and license	7.7%	4
Has no personal transportation, but knows friends/family or other resources that will help them	30.8%	16
Needs transportation assistance	50.0%	26
**Career**		
Has employment lined up for after release	17.3%	9
Cannot work; has/needs SSI (disability)	13.5%	7
Needs assistance finding employment	40.4%	21
Mentioned long-term career goals	17.3%	9
**Education**		
No goals discussed	42.3%	22
GED goal	17.3%	9
Certification or licensure goal	9.6%	5
College goal	26.9%	14

* *Note*: Beginning in October 2021, peers began asking about MOUD specifically in addition to asking about participants’ general interest in OUD treatment of any kind, and asking about children specifically in addition to asking about family relationships in general (n = 29).

#### Recovery

When discussing recovery *supports*, over half of participants (53.8%) mentioned family members, while 15.4% mentioned that they had “good supports,” though no specific individuals were mentioned. Others mentioned relationships with sober friends, sponsors, or employers as recovery supports, yet 13.5% indicated that they had “no support” from anyone. When asked about *mutual support meetings*, most participants (73.1%) mentioned interest or positive previous experiences with 12-step fellowship groups; only three participants mentioned that they had never attended any type of meeting in the past. Religious or faith-based meetings were mentioned by 7.7%, and 5.8% said they were either unsure about meetings or did not want to attend. One participant specifically mentioned Medication-Assisted Recovery Anonymous (MARA) because other 12-step fellowships “are just not welcoming of MOUD.”

Half of participants (50.0%), when asked about *treatment* after release, mentioned that they would like to attend residential treatment, although the length of preferred residence varied from 30 days to 6 months, with many participants simply stating a preference for “long-term treatment.” Fewer participants mentioned a preference for MOUD (17.5%) or outpatient/intensive outpatient (13.5%) in this section. However, in October 2021, peers began asking about interest in MOUD specifically, in addition to asking about participants’ general interest in OUD treatment of any kind. Of those participants asked about MOUD (*n* = 29), almost half (44.8%) were interested in, or planning to pursue, extended-release naltrexone (Vivitrol®), while close to a third (31.0%) were interested in buprenorphine/naloxone (Suboxone®). Three participants (10.3%) indicated that they had no interest in MOUD at the time of their initial assessment.

#### Health

*Mental health* was a common concern among participants, and 53.8% of women specifically mentioned a desire for mental health medications – either to be assessed to initiate medications after release, or to continue or restart medications they had received prior to incarceration. Almost a quarter (23.1%) said that their mental health was “good” or “stable.” Participants were more positive about *physical health*, with 46.2% mentioning that their health was “good.” An additional 13.5% said their health was “good,” but mentioned minor concerns or areas they wanted to work on in the future (e.g. dentures, weight loss, asthma). Chronic, major, and/or immediate health concerns were mentioned by 30.8% of participants, including kidney stones, broken bones, heart problems, epilepsy, and hepatitis C.

#### Relationships

Most women (57.7%) mentioned relationships with *family* that were primarily “good,” “supportive,” or “getting better;” few women (5.8%) mentioned no family support at all. However, almost a third (30.8%) discussed a complex family dynamic: some supportive relationships, while others “need work” or are “strained” or “broken.” Several women also mentioned family members who were incarcerated or in active addiction. Of women who were asked specifically about *children* (*n* = 29, question added after October 2021), 6 had no children, 14 mentioned children but did not discuss them, and 8 disclosed that their children were not living with them and/or not in their custody prior to incarceration. Regarding *friends*, many women (44.2%) reported having at least one sober and supportive friend in their life, but an even greater proportion (46.2%) said they had “no sober friends” outside of jail.

#### Spirituality

Most women (67.3%) told their peer that they were religious or spiritual in some way, while 15.4% indicated they were “working on it.” Fewer women said that they were “unsure” about their spirituality (7.7%) or not spiritual at all (5.8%).

#### Transportation

Very few women (7.7%) said they had their own car and a valid driver’s license. Almost a third (30.8%) had at least one friend/family member or another reliable resource for transportation. Half (50.0%) mentioned a need for transportation assistance, including help obtaining a vehicle or obtaining/reinstating a driver’s license.

#### Career

When asked about their career prospects or aspirations, 17.3% of women mentioned having a job lined up for after their release from jail, while 13.5% said they were unable to work and were receiving supplemental security income (SSI) for a disability or needed help with obtaining SSI. The largest category of women (40.4%) said they would need assistance finding a job after their release. Nine women (17.3%) mentioned more long-term career goals in response to this prompt.

#### Education

More than half of women discussed some type of goal related to education, including completing their GED (17.3%), obtaining some type of certification or licensure (e.g. for cosmetology or peer support; 9.6%), or attending/returning to college (26.9%).

When asked to select a “primary goal” that they would like to work on after release from jail, over half of women (51.9%) chose treatment for OUD (see Table 2). However, women also selected goals related to more basic needs, including transportation (13.5%), employment (11.5%), and housing (7.7%). An additional 7.7% of women reported a primary goal related to interpersonal relationships (e.g. family and/or children). On the BARC-10, out of a possible range of 10–50, women scored between 25 and 50, with an average score of 38.1.

**Table 2.** Reentry goal setting and recovery capital (N = 52)

	**%/** ***M*** **(** ***SD*** **)**	***n***	Range
**Primary goal from initial session:**			
Substance use disorder treatment	51.9%	27	
Transportation/license	13.5%	7	
Employment	11.5%	6	
Housing	7.7%	4	
Family/children	7.7%	4	
Recovery/abstinence	5.8%	3	
Self/general (“get my life together”)	1.9%	1	
**Brief Assessment of Recovery Capital** (BARC-10) total score^a^	38.1 (7.0)	50	25–50

a BARC-10 possible range=10–50 (2 responses missing; n = 50).

### Qualitative Interviews with Peer Navigators: Perceptions of Challenges and Successes

#### Transitions to the community

In the qualitative interviews, the three peers were asked to reflect on their initial sessions with study participants and to discuss what they saw as women’s most pressing needs when preparing for reentry. All three peers discussed the unpredictability of women’s situations as a major barrier to setting goals: women in jails may not have plans for housing or know what treatment recommendations might be made by courts, or even have a precise anticipated release date. As one peer stated, “…it’s hard to establish a goal with a woman when she’s incarcerated. Because… they don’t know what’s gonna happen… They don’t know if they’re going to treatment. They don’t know if they’re gonna have anywhere to go when they leave.” Another peer observed that this lack of stability or support can contribute to a sense of hopelessness, undermining recovery efforts:

When… someone’s incarcerated and you’re sending them out of jail with nowhere to live, what do you expect them to do? … A lot of their attitudes are, well, you know, I don’t have anything anyway, no one supports me, so… why not use drugs, you know? They have no reason to want to do it any differently.

To address the instability and unpredictability of women’s lives, peers often recommended residential treatment as a unified solution to women’s lack of housing, transportation, employment, and recovery supports. One peer stated, “[Residential treatment is] very beneficial when you’re trying to get back on your feet,” in that “it does allow you to get to Walmart, and get to the grocery store, and to your job, and to meetings until you can build that support group yourself.” Another peer discussed how residential treatment allowed time and space for women to work on themselves, without the added stressors of everyday life:

Maybe I’m biased because it saved my life, but I just, I think that you get to learn so much about yourself, and you get to have help in figuring out what that next move’s gonna be… And you’re not in a rush to do it. You know, like if you’re coming straight out of being incarcerated to the streets, you’re having to rush, thinking like, “what do I do?” And then you get overwhelmed. And what happens when you get overwhelmed? You go back out [and start using again].

For women released to more rural areas with fewer accessible services and programs, transitions to the community may be even more challenging. One peer mentioned frustration at not being able to do more for women in these regions:

There’s not a lot of resources available for women there [rural county], so that’s hard… these women need things, they need help. But when there’s no help to give, all I’ve got is me. All I have is an ear to listen and encouraging words to give. And sometimes that’s just not enough.

Nonetheless, peers in the current intervention believed overall that they were serving as a valuable source of information for participants and noted the benefit of serving as a bridge between jail and whatever came next. As one peer said, “I think one of the biggest things that should be conveyed to them is that, like, treatment is literally a call away.”

#### Intervention challenges

In addition to the reentry challenges described above, peers were also asked to discuss challenges related to aspects of the intervention design.

##### Virtual recovery support

One challenge discussed was the fact that peers’ initial contact with women, the baseline assessment, was conducted over videoconference, whereas two peers mentioned that “an in-person baseline would be the most ideal.” One peer mentioned that she thought women might be suspicious of confidentiality (i.e., whether the conversation might be recorded), but both also discussed the difficulty of building rapport through a virtual format. As one peer stated, “It’s hard to build a relationship of trust with someone when you’re over a Zoom one time and then you’re calling them on phone. It’s hard.” Whereas in-person, peers may have more ability to connect with, encourage, or persuade women about the benefits of OUD treatment and the peer recovery support services they offer, peers found this process more challenging in a remote format, unless women were already recovery-motivated: “they [the women] have to really, really, really, really want something different to engage.” Even after women’s release, one peer discussed the difficulty of providing recovery support from a distance:

I feel like… I can be a great cheerleader over here, you know. I can try to give them the correct contacts and things of that nature. But I’m not able to sit down with these women with [job search websites] pulled up at the computer and help them look and see who’s hiring or help them build a resume to apply for these jobs. I’m not able to, you know, sit with them while they call the courthouse to see what they have to do to get their license… there’s a lot of barriers that come into play there.

##### Stigma against MOUD

The other primary challenge discussed by peers was a widespread and persistent stigma against MOUD, particularly methadone or buprenorphine/naloxone (Suboxone®). Many participants equated use of MOUD with not being truly “abstinent” from opiates; as one peer said, “I just always hear, ‘If I’m gonna be off of drugs, I’m gonna be off of them all.’” This peer also speculated that “the stigma for MOUD runs so deep that these women are almost afraid to tell me that they would consider it… I could be wrong, but I almost think… they think I want to hear that they want to be clean from everything, abstinent from everything.” These beliefs about MOUD are often so entrenched that, as another peer said, “you cannot get them to think a new thought when it comes to [MOUD].” Peers in this study received formal training on sharing any personal experiences with prescribed MOUD and education on each form of MOUD so they could speak to the value and efficacy from both a personal and professional standpoint. However, peers also had to exercise judgment to know when advocating for MOUD (instead of other treatment modalities) would be counterproductive to a trusting and supportive relationship with a woman. “When they’re all-out not interested, I leave it,” said one peer. “Because there’s just no point – like, there’s no point me wasting my breath and there’s no point in making them uncomfortable.”

#### Intervention successes and lessons learned

Finally, peers were asked to describe any successes or lessons learned during the first year of study implementation.

##### Planting a seed

Having had only one session via videoconference while women were incarcerated, it was often challenging for peers to reconnect with women in the community after their release from jail. However, one peer discussed the need to “not get discouraged when somebody doesn’t follow back up with you.” Due to the design of the study, women are screened for OUD and invited to participate, but may not be seeking or even ready for treatment, meaning that peers need to “enter into these conversations with empathy, compassion, and to not be pushy… don’t try to be pushy with these people, because they barely even know if they want to be sober or not.” Rather, this peer described the initial session as the opportunity to “plant that seed” about treatment, recovery, and hope, and “see what happens.”

##### Framing peer services in a harm reduction perspective

Given that the larger study is framed as treatment-focused (including a pretreatment telehealth session with a community MOUD provider), one peer discussed the value of framing their services in a nonjudgmental, accepting light, even if participants did not ultimately initiate or engage in treatment, or even returned to use:

I tried to, myself, always let them know, like, if you get out and you return to use, we are still here to help you… we can provide you with those resources to make it safer for you. And just to let them know that we’re there no matter what… when they know that you’re there then [in active use], then they know where to reach out to when they are ready.

Staying in touch with participants regardless of their circumstances allowed peers to be a reliable resource and a source of hope, even when change took time. “I get to continue to talk to them, continue to encourage them,” said another peer; “keep putting bugs in their ear about treatment and [MOUD] and just keep… pouring into them that life can be different, and I can help you make it different.”

##### Sharing your time and yourself

Despite challenges presented by the virtual session format, peers were largely successful in establishing rapport with participants and attributed this success to being present and engaged with women. As one peer discussed:

I try to spend as much time as I can with them, and I try to get them to speak about themselves three times as much as I speak about myself. That’s usually my equation, because there is no point in me harping at them… Everybody wants to talk about themselves, you know; they want to feel important; they want to feel heard. And so, I try to give them that, and give them that time and respect.

In addition to listening to women’s stories, peers also recognized the importance of sharing their own experiences, to offer empathy and hope:

The most important thing I have learned is that I have to share my story with them, connect with them, and let them know that I have been in those shoes… Letting them know…that you’re there for them, and you’ve been there. You’ve had to navigate all of these things on your own once before, so you have first-hand experience, and it just makes them feel like you’re truly there to help and you’re not just… checking the boxes and sending them on their way and filling out some paperwork. They really, truly feel like you care when you share a piece of yourself.

## Discussion

Although previous studies have indicated value in peer services [6–18], including positioning peers in the linkage facilitator or treatment navigation role [19–21], little research has focused on peer navigation specific to SUD treatment for recently incarcerated women. Findings highlight the breadth of needs reported by women in the present sample prior to release, including transportation, employment, housing, mental or physical health, and complex interpersonal relationships, all of which have been highlighted in previous research on women’s reentry [16] and may pose as major barriers to engagement with OUD treatment and sustaining recovery. For women, who may face additional gendered stigma and discrimination, peers may offer critical linkages to a range of needed services, but also the hope, encouragement, and solidarity to initiate SUD treatment and sustain engagement [10,11,15,16].

Results from qualitative interviews elaborated on the magnitude of women’s needs by emphasizing the unpredictable nature of the jail release process and the potential value of residential treatment programs to simultaneously address housing, transportation, physical/mental health, and recovery supports. However, three out of four experimental recruitment jail sites in the present study were located in nonmetro counties, and many women are released to rural areas with fewer available and accessible services, creating challenges for peers to provide referrals and linkages to care. This finding highlights the value of outreach to build recovery support networks across a broad region, particularly with the expansion of remote or telehealth peer services during the COVID-19 pandemic [32].

Among other challenges of the intervention, peers also discussed the difficulty of establishing a foundation for a long-term relationship with participants through videoconference rather than in-person. Although the recent growth in telehealth recovery support has expanded access and reach of services [32], recent research suggests that many peers may lack the additional resources (e.g. technology) necessary to conduct telehealth-based services, and that telehealth services may require a distinct set of competencies to deliver effectively [33]. Under COVID, many peers report increased engagement with new job duties (e.g. coordination of resources) and less time spent providing individual support or group facilitation [34], suggesting that peer services delivered remotely may require targeted trainings to prepare peers for the challenges and responsibilities of a remote role.

Results of this study also highlight the persistent, well-documented stigma related to MOUD among people with OUD and the recovery community [35–38]. According to Anvari and colleagues [36], working with a peer with an aligned recovery pathway is one way to reduce internalized stigma for participants, and this strategy was used in the design of the current intervention. However, it is also necessary to address the stigma related to MOUD in the broader recovery community. The majority of participants in this study reported interest/positive experiences in 12-step meetings. Peers working in MOUD linkage could prepare their participants for the potential to face stigma in these spaces (e.g, difficulty finding a sponsor, pressure to taper) [39]. Additionally, peers can coach their participants in strategies to mitigate this experience, such as: not divulging their use of MOUD or only sharing with a sponsor; attending different meetings to find one that is more accepting of MOUD; or forming a new group [39]. Finally, it is important for peers to be aware of alternative recovery support meetings that are open to all pathways of recovery (such as SMART Recovery and Medication Assisted Recovery Anonymous) and to be knowledgeable about MOUD policies for residential treatment programs or recovery residences that participants may be referred to.

Despite these challenges, peers also framed their work as uniquely valuable, acting both as a source of referrals/information and as a relatable role model. This perspective aligns with previous research on the importance of peers working at the intersection of substance misuse and criminal justice systems [12–16], but is novel given the intervention’s spotlight on women, use of remote/telehealth peer services, and targeted focus on OUD treatment navigation. Findings suggest that women with OUD experiencing short-term incarceration may be a challenging population to engage in research, given the unpredictability of the release process and breadth of needs. However, these very challenges also highlight the importance of including women with justice system involvement in research to focus on mitigating health disparities in this vulnerable population. Furthermore, leveraging the lived experience of women in recovery to bridge translational gaps between researchers and women with active OUD has the potential to expand recovery support networks and build sustainable pathways between criminal justice and healthcare/SUD treatment systems. As a preliminary program description, based on the first year of study implementation, results may not be generalizable to other similar interventions. However, future peer-based interventions can be informed by the challenges and successes of the current study.
